# The role of properdin in complement-mediated renal diseases: a new player in complement-inhibiting therapy?

**DOI:** 10.1007/s00467-018-4042-z

**Published:** 2018-08-23

**Authors:** Marloes A. H. M. Michels, Elena B. Volokhina, Nicole C. A. J. van de Kar, Lambertus P. W. J. van den Heuvel

**Affiliations:** 1 Radboud Institute for Molecular Life Sciences, Department of Pediatric Nephrology, Amalia Children’s Hospital, Radboud University Medical Center, Geert Grooteplein Zuid 10, PO Box 9101, 6525 GA Nijmegen, The Netherlands; 2 Department of Laboratory Medicine, Radboud University Medical Center, Geert Grooteplein Zuid 10, PO Box 9101, 6525 GA Nijmegen, The Netherlands; 3 Department of Pediatrics/Pediatric Nephrology and Department of Development & Regeneration, University Hospitals Leuven, Herestraat 49, 3000 Leuven, Belgium

**Keywords:** Complement system, Properdin, C3 glomerulopathy, Atypical hemolytic uremic syndrome, Proteinuria-induced tubulointerstitial injury, Complement-inhibiting therapy

## Abstract

Properdin is known as the only positive regulator of the complement system. Properdin promotes the activity of this defense system by stabilizing its key enzymatic complexes: the complement alternative pathway (AP) convertases. Besides, some studies have indicated a role for properdin as an initiator of complement activity. Though the AP is a powerful activation route of the complement system, it is also involved in a wide variety of autoimmune and inflammatory diseases, many of which affect the kidneys. The role of properdin in regulating complement in health and disease has not received as much appraisal as the many negative AP regulators, such as factor H. Historically, properdin deficiency has been strongly associated with an increased risk for meningococcal disease. Yet only recently had studies begun to link properdin to other complement-related diseases, including renal diseases. In the light of the upcoming complement-inhibiting therapies, it is interesting whether properdin can be a therapeutic target to attenuate AP-mediated injury. A full understanding of the basic concepts of properdin biology is therefore needed. Here, we first provide an overview of the function of properdin in health and disease. Then, we explore its potential as a therapeutic target for the AP-associated renal diseases C3 glomerulopathy, atypical hemolytic uremic syndrome, and proteinuria-induced tubulointerstitial injury. Considering current knowledge, properdin-inhibiting therapy seems promising in certain cases. However, knowing the complexity of properdin’s role in renal pathologies in vivo, further research is required to clarify the exact potential of properdin-targeted therapy in complement-mediated renal diseases.

## Introduction

In the last decades, it has become evident that the complement system is involved in various diseases [1]. Consequently, the interest in complement-inhibiting therapy has grown immensely [2]. In principle, the complement system is a “good guy” as it is a crucial part of our innate immunity. It consists of numerous plasma and cell surface proteins that strongly interact with each other and with other regulatory (immune) systems to discriminate between foreign, altered-self, and healthy self-surfaces. In this way, complement provides the body with sophisticated immune surveillance and maintenance of homeostasis [3]. An arsenal of complement regulatory proteins keep the complement activity in control and limited to target cells. However, when the balance between the activation and regulation is disturbed, the widespread functions of complement can cause damage to healthy tissues. The kidneys are specifically vulnerable to such injury [1, 4]. In complement-mediated diseases, the blocking of complement-induced damage may outweigh the possible risks of (partly) compromising immunity. Complement inhibition can be focused at different levels of the complement activation cascades. In general, drugs can be grouped into three major functional categories: preventing complement initiation, dampening complement amplification, and blocking the complement effector molecules. Importantly, complement-inhibiting therapy must be considered with care and for each disorder, or maybe even for each patient, individually. This requires a thorough understanding of the pathological mechanisms underlying disease and of the exact functions of the molecule of target [2].

An upcoming but for a long time ignored candidate target is properdin. This complement regulator has a crucial role in promoting one of the three activation routes of the complement system, namely the alternative pathway (AP). The AP is an important contributor to overall complement activity as this pathway has a guarding function providing continuous low-level complement activity and an amplifying function that augments complement activity initiated by all three activation pathways. Properdin is well-known as the only positive regulator of the AP. However, compared to the many negative regulators that attenuate AP activity, our knowledge of the exact functions of properdin in health and disease is lagging behind. Nonetheless, properdin has regained interest in the scientific community, especially in the last 10 years, and this has initiated major advances in the complement field. In this review, a general overview of the basics of properdin biology is given first, covering the production and structure of properdin, its function in the AP, and its clinical significance. Afterwards, the possible role of properdin in renal diseases associated with complement dysregulation is examined, with focus on C3 glomerulopathy, atypical hemolytic uremic syndrome, and proteinuria-induced tubulointerstitial injury. Finally, the potential of properdin as a new therapeutic target for treatment of these diseases is discussed.

## The production and structure of properdin

The complement protein properdin is a highly positively charged plasma glycoprotein. In contrast to most complement components that are being produced by the liver, properdin is mainly synthesized by leukocytes, including T cells [5], monocytes [6], and mast cells [7]. The protein is also stored in the secondary granules of neutrophils from which it is released upon stimulation [8]. The contribution of neutrophils to the total protein levels found in plasma is highlighted by the association between chemotherapy-induced neutropenia and a decline in circulating properdin levels [9]. Normal systemic levels of human properdin generally cover a large range in the healthy control group and are reported in a range from 5 to 45 μg/ml [10–24]. The large range observed among studies is likely due to differences in the methods and reagents used, e.g., detection methods, antibody combinations, and preparations used as the standard protein. Of note, systemic properdin levels are lower in healthy neonates and infants (i.e., < 1 year old) compared to adults [19, 20, 23–28].

**Fig. 1:**
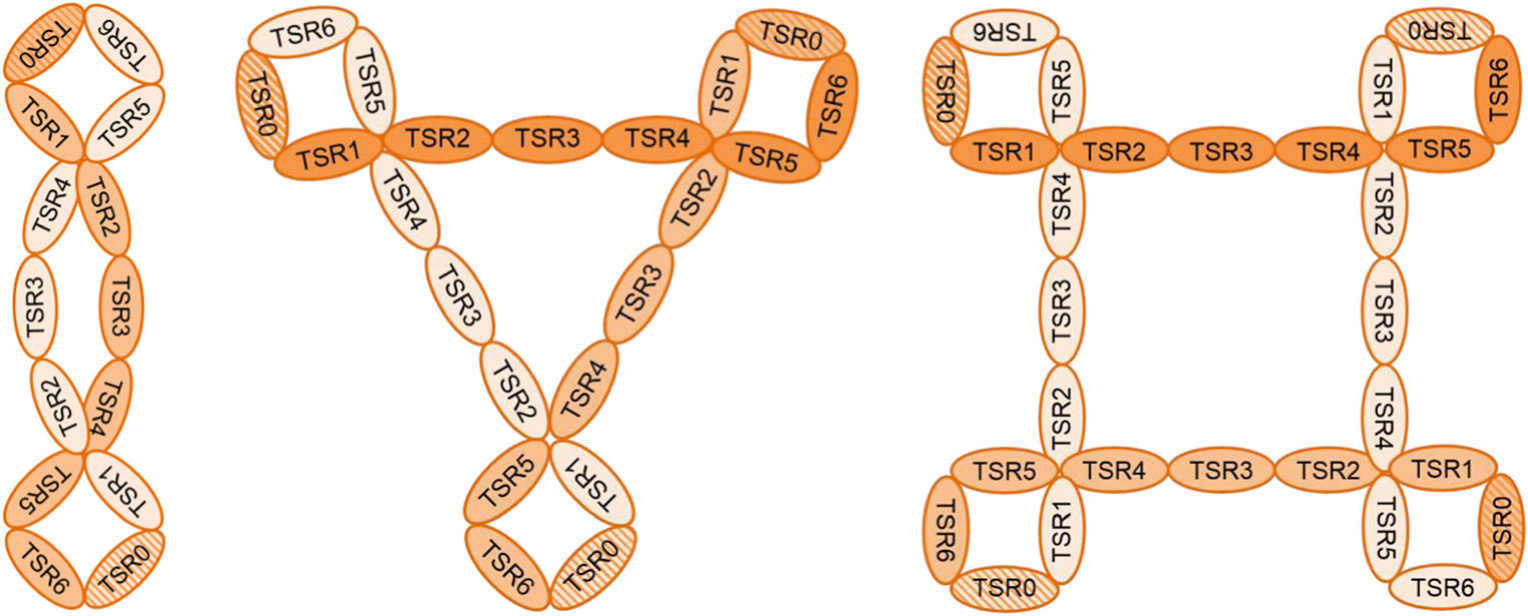
Proposed model of the structure of properdin oligomers. Properdin is composed of monomeric subunits that associate together to form dimeric, trimeric, or tetrameric oligomers with curly vertex structures. Each monomer contains six full thrombospondin type I repeat (TSR) domains named TSR1–6 and a putative truncated N-terminal TSR (indicated with the striped pattern) denoted as TSR0. This latter TSR contains all six conserved cysteine residues although overall sequence homology with the other TSRs is low

Properdin is encoded on the X chromosome and circulates in the blood in oligomeric forms. This latter characteristic is very important for its biological function. The properdin oligomers are composed of identical rod-like monomers of ~ 53 kDa [29]. Each monomer consists of 442 amino acid residues [30] and comprises one presumed truncated and six full thrombospondin type I repeat (TSR) domains, numbered TSR0 to TSR6 from the N to C terminus [30–32]. The flexible monomeric subunits associate head-to-tail into mainly cyclic dimers, trimers, or tetramers with curly vertex structures (Fig. 1) [10, 29, 33]. In normal human plasma, the oligomers exist in a fixed ratio of approximately 1:2:1, with the trimer being the predominant form [10].

The flexible, oligomeric nature of properdin is challenging for structural and biochemical studies. For a long time, information about the functions of the individual TSRs regarding target binding and oligomerization had therefore been derived primarily from structure-function studies using mutated/truncated recombinant proteins or specific TSR-directed antibodies [31, 34, 35]. Recent advances in the elucidation of the atomic structure of properdin have now indicated that four TSR domains, originating from two monomers, are involved in vertex formation. This leaves three TSRs to form the connecting part [33, 36]. The most likely composition of properdin, based on current knowledge, is a vertex with TSR0–1/TSR5–6 connected by TSRs 2–4 (Fig. 1). A complete, high-resolution atomic structure of properdin (in complex with the convertase) still needs to be resolved.

## The function of properdin in the alternative pathway of the complement system

### Activation of the alternative pathway

**Fig. 2:**
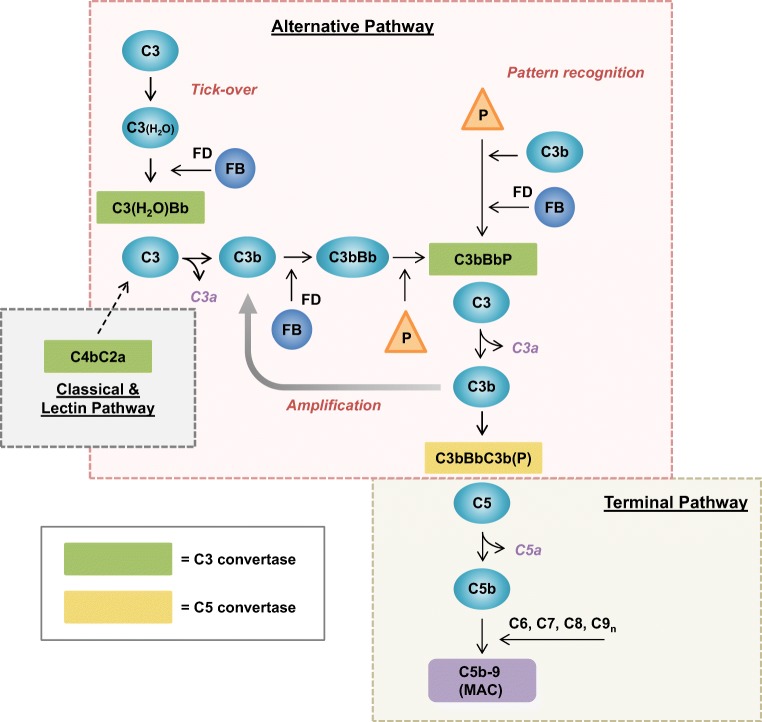
The alternative pathway of the complement system. Activation of the complement system can be achieved via three pathways—the classical, lectin, and alternative pathways—depending on the trigger that is recognized. The proteolytic enzyme cascades of these three pathways converge at the central event of complement activation, which is the cleavage of C3 into C3a and C3b by C3 convertases. The classical and lectin pathways are activated using pattern recognition molecules that initiate formation of the C3 convertase C4bC2a. The alternative pathway is continuously activated at a low rate by a mechanism called “tick-over.” The spontaneous hydrolysis of C3 generates the active C3(H_2_O) fragment which can form an initial fluid-phase C3 convertase upon association with factor B (FB) that is subsequently activated by factor D (FD) into Bb. Under normal conditions, this convertase generates small amounts of activated C3 fragments. C3a is released as an anaphylatoxin to mediate inflammation, for instance by attracting leucocytes. C3b is an opsonin; it binds to molecular or cellular target surfaces and marks them for phagocytosis. C3b can also act as a platform for formation of new C3 convertases which are effectively stabilized by properdin (P). The alternative pathway may also be initiated by properdin as this molecule recognizes target surfaces and subsequently recruits C3b and FB to form stabilized C3 convertases. Alternative pathway C3 convertases are important amplifiers of the complement reaction by converting many C3 molecules into C3b which in turn support new C3 convertase formation. Furthermore, C3b can attach to preformed C3 convertase complexes to form C5 convertases that convert C5 into C5a (an anaphylatoxin similar to C3a) and C5b to initiate terminal pathway activity. The C5b activation fragment recruits a series of other complement components, i.e., C6, C7, C8, and multiple C9 molecules, to form the membrane attack complex (MAC; C5b-9). This protein complex forms a pore that disrupts the membrane integrity and thereby can cause osmotic lysis of susceptible bacteria and cells. In sublytic amounts, MAC causes cell damage by activating still not well-understood (pro-inflammatory) signaling pathways

Properdin promotes complement activity by specifically acting on the AP. In contrast to the classical (CP) and lectin (LP) complement activation pathways, the AP is constantly active at a low level under normal conditions. Spontaneous hydrolysis of C3 at a low rate enables formation of an initial, fluid-phase C3 convertase (C3(H_2_O)Bb) that is able to cleave C3 into its active components C3a and C3b (Fig. 2). This mechanism is known as “tick-over” and generates a persistent low level of activated C3 molecules that provide the host with constant responsiveness to potential danger. C3a is released as an anaphylatoxin to mediate inflammation, while C3b marks target cells near its activation site for phagocytosis in a process known as opsonization. Activated C3b molecules can also be generated by the activity of C3 convertases from the other two activation pathways that are formed upon recognition of immune complexes or specific carbohydrate signatures. Such pattern recognition function has also been assigned to properdin for the AP in certain contexts (see “Properdin as an initiator of alternative pathway activity” for more details). Once complement is initiated and active C3b has been formed, the AP supports an important amplification loop. By interacting with factor B (FB), factor D (FD), and properdin, target-bound C3b can form effective, stabilized AP C3 convertases (C3bBbP). These surface-bound convertases can amplify the complement response by converting many more C3 molecules into C3b, which in turn support new C3 convertase formation, C5 convertase formation, and the initiation of further downstream effects of terminal pathway activation (see Fig. 2) [3, 4, 37]. C5 convertases convert C5 into C5a, which is a strong anaphylatoxin, and C5b. This latter fragment forms the base for the assembly of the membrane attack complex (MAC; C5b-9). This pore-forming protein complex is inserted into membranes to induce direct osmotic lysis of the target cell or indirect cytotoxicity via pro-inflammatory pathways [3, 4, 37]. Thus, besides being an important immunosurveillance system, the AP is also an important amplifier of initiated immune responses as it can account for over 80% of total complement activity [38].

### Regulation of alternative pathway activity

The forceful activity of the AP must be tightly regulated to avoid damage to healthy host cells and tissues. Several cell-bound and soluble complement regulatory proteins keep AP activation restricted to target surfaces and keep excessive AP activation in control [3, 4, 37]. The regulatory mechanisms are mainly aimed at inhibiting C3 convertase activity and can be divided into two ways of action. First of all, complement regulators may have cofactor activity for the circulating complement regulator factor I. This soluble protease cleaves C3b into inactive fragments that no longer support convertase formation. Secondly, regulators may have decay-accelerating activity, which means they promote the dissociation of the convertases. Regulators with this activity may also compete with FB to bind to C3b and thereby prevent convertase (re-)formation. Factor H (FH) is the most abundant and important soluble regulator. It has all of the abovementioned activities and can control AP activation both in fluid phase and on surfaces to which it is recruited, also on tissue structures that do not express membrane-bound regulators. Properdin is the only known complement regulator that promotes instead of inhibits AP activity. In addition, properdin only interacts with AP convertases, in contrast to some of the other AP complement regulators that also regulate CP/LP convertase activity [3, 4, 37].

### The place of properdin in the alternative pathway

Initially, when the AP was still to be discovered, properdin was thought to be the initiator of serum-dependent complement activity in the “properdin system” defined by Louis Pillemer in 1954 [39]. His work showing a non-antibody-dependent way of complement activation was very controversial at that time and was difficult to reproduce; hence, it received much criticism. After the elucidation of the “tick-over” mechanism as an initiator of AP activity, this initiating function of properdin was replaced by its well-known function as a stabilizer of AP convertases [40]. Nonetheless, biochemical studies in the last two decades have provided new evidence for a function of properdin in directing and triggering complement activation on potentially dangerous targets.

#### Properdin as a stabilizer of alternative pathway convertases

Under physiological conditions, convertases are unstable enzyme complexes with a short half-life of around 90 seconds [41, 42]. In association with properdin, their stability increases up to tenfold [42, 43]. Properdin forms particularly strong interactions with the C-terminal part of C3b and binds as well to Bb [33, 36, 44, 45]. Its affinity for C3bB (the pro-convertase) and C3bBb complexes is much higher than that for C3b alone [44, 46]. Thus, properdin might stabilize these complexes by holding the two components of the convertase together. Besides, its binding induces a conformational change in C3b, which hinders both the decay-accelerating and cofactor activity of complement regulators, such as FH [33, 36, 44]. As such, properdin is an important positive regulator of the AP.

**Fig. 3:**
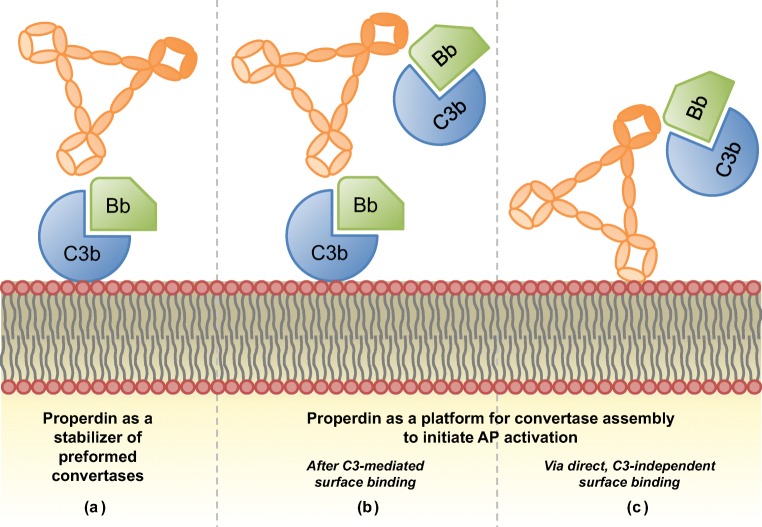
The functions of properdin in the alternative pathway. The interactions of properdin, depicted in its trimer form, with C3 convertases (C3bBb) and a surface are displayed. (A) Properdin as a stabilizer of preformed convertases on a surface. (B, C) Properdin as a platform for convertase formation after initial C3-mediated binding (B) or as a pattern recognition molecule by directly recognizing target surface structures (e.g., glycosaminoglycans and exposed DNA) and subsequently recruiting convertase components (C3bBb, C3bB, or C3b and FB)

From the structural studies of Alcorlo et al. [33] and Pedersen et al. [36], it became clear that the vertexes of properdin are responsible for this binding and stabilization of convertases (Fig. 3(A)). Oligomerization of at least two monomers to form these vertex structures is therefore essential for properdin’s function in vivo [33, 36]. In addition, it was shown that oligomers can use each of their vertexes for convertase binding [33, 46]. This may explain why tetrameric and trimeric properdin are reported to be more active than dimeric properdin [10, 47].

The stabilizing action of properdin is focused predominantly on surface-bound convertase complexes, as the affinity of properdin for surface-bound C3 derivatives is much higher than that for fluid-phase fragments [44]. In line with this, it is supposed that properdin facilitates the switch from the C3 to the C5 convertase on surfaces [48]. C5 convertases are only efficiently formed on surfaces, and not in fluid phase, since their formation requires high densities of C3b [49]. There are strong indications that absence of properdin is associated with reduced C5 convertase and terminal pathway activity (also see “Unexpected findings of properdin gene knockout in C3G”) [11, 48, 50–56]. It remains unclear, however, if properdin is strictly needed for C5 convertase formation and/or if properdin stabilizes the C5 convertase complex (C3bBbC3b) itself. This latter function is generally assumed based on the high similarity between C3 and C5 convertases, and it is often stated in literature with references to studies performed in the 1970s and 1980s [42, 57, 58]. In our opinion, convincing up-to-date evidence has yet to come.

#### Properdin as an initiator of alternative pathway activity

In 2006, Hourcade published a study that renewed the interest in the concept of properdin as an initiator of AP activity [46]. It was demonstrated that properdin could not only stabilize C3bBb complexes but also actively accelerate the association of C3b with FB to form C3 convertases. More importantly, properdin that was bound to an artificial surface, either directly or via C3 convertase intermediates (C3b, C3bB, or C3bBb), was able to recruit components from the environment to form new convertases on its unoccupied binding sites. Thus, properdin could serve as a platform for de novo convertase assembly on a surface (Fig. 3(B, C)) [46]. Numerous studies followed which extended these findings to biological surfaces under more physiological conditions. Targets on which properdin could initiate AP activity included typical AP targets (e.g., zymosan and rabbit erythrocytes), dangerous non-self surfaces (e.g., *Chlamydia pneumoniae*), altered-self surfaces (e.g., apoptotic and necrotic cells), and also self surfaces such as platelets and proximal tubular epithelial cells (PTECs) [7, 17, 59–69]. Suggested binding ligands on non-microbial cells are glycosaminoglycans [60] and surface-exposed DNA [17]; both are negatively charged.

Whether endogenous properdin in serum can indeed act as a true pattern recognition molecule in the meaning of being able to recognize and *directly* bind surface structures without primary C3b deposition in vivo (Fig. 3(C)) is a subject of controversy. The conflicting findings on this topic depend on the following three main aspects: (1) the source of properdin that is used, e.g., purified from serum, in whole serum environment, or freshly secreted from leukocytes; (2) the experimental conditions under which the assays are performed, e.g., allowing initial C3b deposition or not; and (3) the choice of biological target surface studied. For instance, caution should be taken in interpreting results obtained through the use of purified properdin, as it can form large polymeric aggregates, especially upon freeze-thaw cycles [10, 70]. These non-physiological aggregates have different AP-activating and target-binding properties than the physiological properdin oligomers that were separated from these polymeric aggregates after purification [10, 63, 64, 67, 70]. In addition, to confirm that properdin is a true recognition molecule, conditions should be used in which no initial C3b deposition can take place [17, 71, 72]. Some studies have suggested that circulating properdin in C3-inactive serum was not able to bind freely to certain targets which purified physiological properdin oligomers [64–67, 72] or properdin freshly released from activated leukocytes [60] *could* bind. C3-inactive serum refers to serum in which C3 (activation) is blocked or removed, for example by adding a C3-blocking molecule, using C3-depleted serum, or using a calcium- and magnesium-chelating agent to prevent complement activation. These findings have led to the hypothesis that serum contains inhibitors that prevent or regulate the direct pattern recognition function of properdin in the circulation [73, 74]. Such a regulatory mechanism could serve to prevent unwanted properdin-mediated damage systemically and to keep the pattern recognition preserved for specific conditions of danger or disease. In a recent study of O’Flynn et al., monomeric (but not pentameric) C-reactive protein was identified as such a properdin-regulating molecule, as it was able to control properdin-mediated AP activation on the PTEC surface [75].

In conclusion, it is evident that properdin can act as a docking station for convertase assembly and further AP activation (Fig. 3(B)). However, confirming its potential as a direct pattern recognition molecule in different in vivo environments (Fig. 3(C)) demands for a critical evaluation of used properdin preparations and experimental setup. Taking these conditions into account, the most important properdin-target interactions published so far can be summarized as follows: purified physiological properdin oligomers were shown to bind directly to zymosan [63, 64], necrotic, diseased B and T cell lines [64], *Chlamydia pneumoniae* [65], and activated platelets [67]; freshly secreted properdin was found to bind to apoptotic T cells [60] and activated platelets [67]; and properdin in C3-inactive serum has only been shown to bind to necrotic cells so far [17]. Interestingly, recent indications of other possible AP activators have emerged, such as complement FH-related protein 4 [76] and FH-related protein 5 [77, 78]. Comparable pattern recognition and AP-activating and enhancing functions were attributed to these molecules as to properdin, but these proteins also need further investigation as to their exact role in different physiological contexts.

### Properdin in the local microenvironment

Besides a systemic role in complement, properdin can also act as an important and powerful mediator in (pro-inflammatory) local microenvironments. At these sites, properdin-producing cells are abundant and the properdin they produce might escape the serum inhibition that is assumed to exist in the circulation. Thus, locally strong increases in properdin levels may be generated which may modulate diverse immune responses at these sites (reviewed in [79]). First of all, properdin may have an important role in the safe and effective clearance of dying/dead cells. Direct binding of freshly secreted properdin from neutrophils to apoptotic and necrotic cells may aid in the phagocytosis of these cells [60]. Besides, the high amounts of properdin released as a response to local stimuli may amplify the AP-mediated inflammatory events in the microenvironment. Properdin-mediated complement activation contributes to further recruitment of pro-inflammatory cells to the site of infection via generation of the potent neutrophil chemoattractant C5a. In turn, neutrophils are triggered to release properdin from their granules, which may further act in a positive feedback loop by activating complement at its own surface and by activating and recruiting more neutrophils to inflammation sites [79, 80].

## Clinical significance of properdin

### Properdin deficiency

Properdin deficiency is a rare X-linked disorder that mainly affects males and is strongly associated with an increased vulnerability for meningitis caused by *Neisseria meningitidis* strains [81, 82]. Over 100 cases have been documented in over 25 families [82, 83]. Compared to properdin-normal individuals, meningococcal infections in properdin-deficient patients are associated with higher mortality and are more frequently caused by uncommon serogroups. Besides, the infections generally occur during teenage years instead of during early childhood as in the general population [84–86]. In addition to the increased risk for meningococcal infection, a recent study found that in one family, properdin deficiency was associated with recurrent otitis media and pneumonia [87]. These findings underline the importance of properdin in the host defense against certain but apparently not all pathogens.

Three types of properdin deficiency are recognized: type I describes the complete absence of the protein and is the most common, type II encompasses the cases in which levels are very low (up to 10% of normal), and in type III, systemic properdin levels are normal, but the protein is functionally defective [82]. Female carriers present with on average half of the normal properdin levels; levels range from nearly zero to concentrations in the normal range due to an uneven inactivation of the mutated and normal X chromosome [84, 88]. The mutations underlying properdin deficiency are very heterogeneous. In type I deficient families, various nonsense and missense mutations and few small deletions have been characterized [82, 88–91]. These were located in exons 4 to 9, encoding TSR1–5 and the first part of TSR6, respectively [82, 88–91]. The genetic changes in type I deficiency typically affected highly conserved amino acids [82] and are expected to alter the conformation and/or stability of properdin in such a way that it cannot be excreted anymore and becomes catabolized intracellularly [92]. The three underlying genetic defects found in type II deficiency are two missense mutations, one in exon 4 [93] and one in exon 8 [92], and one small combined deletion/insertion in exon 5 causing a frameshift and premature stop in exon 7 [87]. As a result of these mutations, properdin is likely impaired in its oligomerization, but the effect on structure is probably less drastic than expected in type I deficiency. Rapid catabolism of abnormal properdin molecules extracellularly might explain the low systemic properdin levels [92]. Only one family with type III deficiency has been identified [94]. The affected Dutch family members contained a missense mutation in exon 9 that likely affected C3b binding by properdin [95].

### Potential role for properdin in human pathologies

The involvement of properdin in other human disease settings has only recently started to become elucidated. In the last decade, multiple studies to (complement-related) diseases have emerged in which alterations in systemic properdin levels were found (see Table 1). These studies have given us an indication of diseases in which properdin might play a significant role. Importantly, as in healthy controls, properdin levels in the studied patient cohorts generally also cover a large range. Reduced systemic properdin levels as compared to controls were observed in various disease conditions, such as in the renal diseases C3 glomerulopathy (C3G) and lupus nephritis [9, 11–14, 16, 21, 96] (Table 1). This may indicate that properdin is “consumed” in the circulation due to high AP activity; i.e., properdin is repositioned from the bloodstream to complement convertases at local sites of (surface-bound) complement activation to drive the AP activity. Lowered concentrations in the blood may also indicate a problem with the properdin-producing cells as in neutropenia [9]. Few studies also found increased systemic properdin levels (Table 1), for example in IgA nephropathy and in patients on hemodialysis [15, 18, 22, 97, 98]. The observation that increased properdin levels were associated with cardiovascular events and reduced levels with chronic heart failure was somewhat contradicting and needs further examination [16, 98]. A possible explanation might be that the role of complement changes at different stages of a disease. At the initial phase, elevated properdin levels might mainly increase the risk for developing the full disease. Once the disease has advanced and is clearly manifested, properdin levels might be reduced due to consumption [16]. Furthermore, some studies have reported pathological conditions in which clear local changes in properdin were found. For example, properdin levels increased in the bronchoalveolar lavage of allergic asthma and rhinitis patients after allergen challenge, which may indicate increased production/infiltration of properdin-producing inflammatory cells [99]. Future research will define whether more AP-mediated (renal) diseases show disturbances in properdin levels systemically and/or locally or whether this is selective for certain disease mechanisms only. After all, in some AP-related disease groups, no alterations in systemic properdin levels could be discovered [100].

**Table 1:** Overview of human diseases and conditions associated with altered systemic properdin levels

Disease/condition	Findings	Reference
a. Diseases and conditions associated with *reduced* systemic properdin levels		
C3 glomerulopathy	Reduced P levels compared to controls. Average P levels were almost two times lower in C3GN compared with DDD, while sC5b-9 levels were elevated in C3GN compared with DDD.	Zhang et al. 2014 [12]
	Reduced P levels compared to controls (i.e., below the mean-2sd) in 53% of the patients negative for C3NeF. C3GN was more frequent in the C3NeF-negative group, but no difference in C3GN frequency between the groups with normal versus reduced P. P consumption correlated with reduced C3 and C5 levels, with elevated sC5b-9 levels, and with a higher degree of proteinuria.	Corvillo et al. 2016 [11]
	Reduced P levels just below the lower limit of the reference range of controls in 4 out of 5 patients positive for C4NeF. Also decreased serum C3 and C5 levels, while C3c and sC5b-9 were increased.	Zhang et al. 2017 [13]
Anti-neutrophil cytoplasmic antibody-associated vasculitis	Reduced P levels in active phase versus controls and versus remission, while plasma C3a, Bb, C5a, and sC5b-9 were elevated in active stage compared to remission. P levels inversely correlated with the proportion of crescents in the renal specimen.	Gou et al. 2013 [21]
Lupus nephritis	Approximately two-times reduced P levels in active lupus nephritis compared to controls, accompanied by increased plasma C3a, Bb, C5a, and C5b-9.	Gou et al. 2013 [21]
Human sepsis	Reduced P levels in patients on admission to the intensive care unit compared to controls. Slightly lower P levels in non-survivors compared to survivors. Low P levels correlated to increased treatment duration.	Stover et al. 2015 [14]
Chronic heart failure	Reduced P levels compared to controls, especially in those with a more advanced clinical disease, while FD and sC5b-9 were increased. P levels correlated with measures of cardiac function and were associated with adverse outcome.	Shahini et al. 2017 [16]
Viral lower respiratory tract infections	Reduced P levels in patients with severe compared to mild disease^a^, although no differences found in acute versus recovery samples.	Ahout et al. 2017 [96]
Chemotherapy-induced neutropenia	Reduced P levels in the neutropenic state versus the preneutropenic state with normal neutrophil counts.	Tsyrkunou et al. 2017 [9]
b. Diseases and conditions associated with *increased* properdin levels		
Healthy first-degree relatives of type 2 diabetes subjects	Elevated P levels in healthy first-degree relatives of type 2 diabetes subjects compared to age-matched controls. FB and sC5b-9 were also significantly higher in first-degree relatives, but no differences in C3, Bb, C3a, or FH.	Somani et al. 2012 [15]
Hemodialysis	Elevated P levels (by approximately factor 1.3) compared to controls, and slightly higher levels at the end of the hemodialysis session compared to the start. Also increased levels of C3d and C5b-9 after hemodialysis.	Poppelaars et al. 2016 [18]
Antibody-mediated rejection in heart transplant recipients	Elevated P levels in AMR patients carrying a rare AMR-associated allele in the P gene compared to control patients not carrying the rare allele and without AMR.	Marrón-Liñares et al. 2017 [97]
Cardiovascular events	Elevated P levels were associated with endothelial dysfunction, and with the risk of cardiovascular events.	Hertle et al. 2016 [98]
IgA nephropathy	Elevated P levels (by approximately factor 1.5) compared to controls. Also in the patients followed over time, P levels remained higher.	Onda et al. 2007 [22]

^a^No data on age-matched controls in this study involving very young children and no correction for age between the disease groups

*P* properdin, *C3GN* C3 glomerulonephritis, *DDD* dense deposit disease, *C3NeF* C3 nephritic factor, *C4NeF* C4 nephritic factor, *sC5b-9* soluble C5b-9, *FD* factor D, *FB* factor B, *AMR* antibody-mediated rejection

### Studies of properdin-deficient mouse models

Studies using properdin-deficient mouse models or mice treated with anti-properdin antibodies have provided us with indications about the beneficial and detrimental outcomes of properdin-inhibiting strategies. In general, the absence of properdin has been found to be beneficial in mouse models in which the complement activation is directed to host cells or tissues. Elimination of properdin has been shown to be promising in mouse models of renal ischemia-reperfusion injury [101, 102], arthritis [52, 53, 103], allergic airway inflammation [99], abdominal aortic aneurysm formation [104], and, very recently, atypical hemolytic uremic syndrome (aHUS) [105]. Properdin inhibition has also been shown effective in preventing complement-mediated extravascular hemolysis [62] and embryonic lethality [103] induced by the absence of an important membrane-expressed murine complement regulator. The better outcome of properdin-affected mice in these disease models is likely due to abrogated/attenuated AP activation and thus alleviation of the injuring immune responses directed to host tissues. On the contrary, in cases of infection where complement is directed to the invading pathogen, absence of properdin was often found detrimental for the host. Properdin deficiency has been associated with an exacerbated disease outcome in models of polymicrobial septic peritonitis [7], colitis [50, 51], small intestinal mucositis [106], LPS-induced non-septic shock [107], and *Listeria*-induced septicemia [108]. In these cases, properdin-deficient mice likely had a detrimental outcome due to compromised host defense against the microbial intruder, indicating properdin played a crucial role in this process. Nonetheless, the abovementioned concepts to predict outcome of properdin blockage do not always hold true. Whereas properdin-deficient mice indeed showed reduced survival in the model of non-septic shock induced by LPS, properdin-deficient mice were more resistant to zymosan-induced non-septic shock compared to wild-type mice [107]. In addition, in murine septicemia models, properdin deficiency worsened the outcome when the disease was induced by an infection of *Listeria monocytogenes*, whereas it improved the outcome when the pathology was induced by *Streptococcus pneumoniae* infection [108]. Also unexpected were the findings that absence of properdin in C3G, a disease characterized by glomerular injury due to excessive (fluid phase) AP activation, resulted in exacerbated renal injury [54, 109]. These studies indicate that there is a complex interplay between properdin and other (immune) effectors acting at the site of injury which determines what outcome the absence of properdin will trigger.

## The role of properdin in complement-mediated renal injury

Genetic variations in complement genes and/or the presence of autoantibodies changing the function of complement components may disturb the sophisticated balance of AP activation and regulation. Especially in combination with triggering events, this may result in an overactivation of the system with subsequent damage to healthy tissues. The glomerulus is particularly vulnerable for complement attack; AP dysregulation has been associated with the disease entity C3G and with aHUS [110–115]. Besides, the renal tubular system can become prone to complement activation in diseases accompanied with proteinuria [116, 117]. The following section focuses on the role of properdin in developing these complement-associated pathologies.

### C3 glomerulopathy

#### Clinical manifestation and pathogenesis

C3G describes a spectrum of severe complement-mediated renal diseases with up to 50% of patients progressing to end-stage renal disease within 10 years after first presentation [48, 115, 118–123]. C3G is characterized by the accumulation of C3 breakdown fragments in the glomeruli, without or with sparse immunoglobulin deposition [118, 124]. The causative disease process is an abnormal control of complement activation, deposition, or degradation [124]. Patients often present with low serum C3 levels as a result of the enhanced C3 turnover in the fluid phase. The main two diseases encompassed by the disease entity are dense deposit disease (DDD) and C3 glomerulonephritis (C3GN), which are distinguished from each other based on electron microscopy appearance. DDD is diagnosed on renal biopsy based on the presence of very dense ribbon-like intramembranous deposits in the glomerular basement membrane (GBM), while in C3GN these deposits appear in a less dense, more amorphous, and more diffuse pattern [124]. The most important pathogenic factors in C3G are autoantibodies stabilizing the AP C3 convertase, so-called C3 nephritic factors (C3NeF; 40–80% of cases), although pathogenic variants in AP (regulating) genes have also been reported (~ 20% of cases) [114, 115, 123]. Both C3NeF and properdin stabilize AP C3 convertases, but whereas C3NeF is strongly associated with pathogenic conditions and indicates a dysregulation of complement activity, properdin is part of healthy homeostasis and has a complement regulatory role.

#### Unexpected findings of properdin gene knockout in C3G

As C3G occurs as a result of overactivation of the AP leading to glomerular injury, it was hypothesized that inhibition of properdin could prevent this AP overactivity and subsequent injury. Surprisingly, however, mouse models showed a protective role for properdin in C3G (summarized in [125]). The mouse models used were FH-deficient mice (FH^−/−^) [54] or mice with only small amounts of truncated FH (FH^m/m^) [55, 109]. These two models both showed the characteristic accumulation of C3 along the GBM with morphological changes and glomerular inflammation typical for C3G. Besides, these mice had low plasma C3 and C5 levels as a result of complement consumption by the activity of the C3/C5 convertases, and thus clearly show the lack of FH-dependent complement regulation [54, 55, 109]. However, mice that were knocked out for both FH and properdin, FH^−/−^/P^−/−^, showed exacerbated injury with increased accumulation of C3 along the GBM. FH^m/m^/P^−/−^ mice showed a similar detrimental effect of the absence of properdin; the renal injury was even worse since the mice died prematurely of severe glomerulonephritis. Remarkably, both these FH-affected properdin-knockout models showed a selective C3 depletion but higher intact C5 levels (less C5 consumption) compared to the mice with an intact properdin gene [54, 55, 109]. Thus, absence of properdin specifically reduced the C5 turnover and was associated with the exacerbated injury observed in these properdin-knockout mice.

#### Possible role for properdin in the distinction of C3G subgroups with different pathophysiology

The differences in plasma C3 and C5 levels between properdin-deficient and properdin-sufficient mice are reminiscent of the complement profiles found in C3G patient groups associated with two types of C3NeF, namely properdin-dependent and properdin-independent C3NeF (Table 2) [48, 126–130]. C3NeF are a heterogeneous group of autoantibodies. All stabilize the AP convertases by preventing intrinsic and/or extrinsic decay [130], but some of them have the ability to induce both systemic C3 and C5 consumption (properdin-dependent C3NeF; resembling properdin-sufficient FH-affected mice), whereas others only seem to affect the levels of C3 (properdin-independent C3NeF; resembling properdin-deficient FH-affected mice) [48, 126–129]. A recent study suggested the new term C5NeF for C3NeF that are dependent on properdin and cause increased C5 conversion [48]. In humans, properdin-dependent types of C3NeF were more often found in C3GN [48, 126, 128]. In this C3G subtype, C5 convertase dysregulation and terminal pathway activation are more pronounced, indicated by lower C5 and higher soluble C5b-9 (sC5b-9) levels in the circulation (Table 2) [12]. Properdin-independent C3NeF are more frequently associated with DDD cases [48, 126, 128]. This subtype is characterized by a selective, more pronounced C3 convertase dysregulation, as shown by lowered C3 levels but near-normal C5 and sC5b-9 levels [12]. Thus, these biomarker profiles of C3G subtypes are in line with the functional characteristics regarding C3 and C5 conversion attributed to the properdin-dependent and properdin-independent C3NeF types.

**Table 2:** Proposed underlying mechanisms in the pathophysiology of C3 glomerulopathy based on the presence of different types of convertase-stabilizing nephritic factors

		
Type of C3NeF	Properdin-independent C3NeF	Properdin-dependent C3NeF/C5NeF
Associated complement profile	C3 consumption	C3 consumption
	C5 normal or slightly consumed	C5 consumption
	sC5b-9 normal	sC5b-9 elevated
Disease association	DDD	C3GN
Comparative mouse model	FH^−/−^/P^−/−^	FH^−/−^

*C3NeF* C3 nephritic factor, *C5NeF* C5 nephritic factor, *sC5b-9* soluble C5b-9, *DDD* dense deposit disease, *C3GN* C3 glomerulonephritis, *FH* factor H, *P* properdin

The biomarker analysis of Zhang et al. also showed direct differences in the level of properdin in C3G patients [12]. Properdin levels were found lower in C3G compared to controls, and the effect was more pronounced in the C3GN group than in the DDD group [12]. Next to the terminal pathway biomarker sC5b-9, properdin was the only AP marker that significantly differed between the two C3G subgroups. Another study also showed significantly lowered serum levels of properdin in a subset of C3G patients, namely in C3NeF-negative patients which were predominantly C3GN cases [11]. Interestingly, the properdin consumption was associated with increased C5 convertase activity leading to C5 consumption [11]. A recent cluster analysis approach performed by Iatropoulos et al. confirmed that different complement dysregulation profiles at the C3 and C5 levels exist in patient subgroups in C3G [131]. However, the identified clusters were not simply divided in C3GN and DDD but were based on shared clinical, histological, genetic, and serological complement parameters to define distinct disease entities characterized by specific pathophysiological mechanisms [131]. Properdin was not taken into account. More research is needed to investigate whether properdin, in combination with different types of nephritic factors, might be the driving force in directing the C3 or C5 convertase dysregulation and subsequent pathophysiology in C3G subgroups, and as such may be an important disease biomarker.

A role for properdin in the pathogenesis of C3G is slightly unexpected in the view of C3G being a disease caused by fluid-phase dysregulation of the AP. How properdin alters the balance of C3 and C5 consumption and how this exactly results in exacerbation of renal injury in the FH-affected mouse models requires further investigation. The current hypothesis is that properdin deficiency changes the ratio between systemic, fluid-phase (properdin-independent), and local, surface (properdin-dependent) AP activation [54–56, 109]. Depending on the presence of properdin and the availability of intact C3 and C5, deposition of systemically generated breakdown fragments and/or local AP activation may be directed to different renal structures, e.g., the endothelium, the unprotected, exposed GBM (on which properdin-independent activation may be possible), the mesangium, or the renal tubules, to cause injury to different extents. In this way, properdin may determine the intraglomerular fate of C3 breakdown fragments which are related to the different C3G subforms [54–56, 109]. Thus, further investigation into the role of properdin in C3G may aid in our understanding of the pathophysiology and distinction of C3G subforms.

### Atypical hemolytic uremic syndrome

#### Clinical manifestation and pathogenesis

Atypical HUS is categorized as a thrombotic microangiopathy and is clinically characterized by the typical triad of hemolytic anemia, thrombocytopenia, and serious renal impairment [132, 133]. These symptoms are caused by uncontrolled AP activation focused on the glomerular endothelial cell surface. Via diverse pro-inflammatory and pro-thrombotic events, this complement activation leads to the disturbed integrity of the endothelial cell layer and subsequent renal injury [112]. In around 60% of the aHUS patients, genetic aberrations have been found in complement genes [134–136]. These aberrations include loss-of-function mutations in genes encoding complement regulatory proteins and gain-of-function mutations in genes encoding the constituents of the convertase complex. The FH gene is most frequently affected [134–136], and FH-directed autoantibodies are also a common cause of impaired complement control [137, 138]. This illustrates the importance of this regulator in protecting the glomerular structures.

#### Possible role for properdin in aHUS

Although many mutations in complement regulators have been associated with aHUS development, little is known about whether properdin may be affected in patients. To our knowledge, no gain-of-function mutations in properdin have been described in patients so far. Also from our own unpublished observations, we can conclude the properdin gene is not affected in aHUS patients. Thus, to date, there is no direct evidence for a role of properdin in aHUS in humans.

Nonetheless, studies with mice have shown that properdin plays a critical role in AP activation on autologous cells. Mice were modeled for disorders in which the surface control on autologous cells was impaired by knocking out important membrane-bound complement regulatory proteins. The subsequent vulnerability of these cells to complement-mediated attack was dependent on the presence of properdin, since properdin-deficient mice showed less injury and better disease outcomes [62, 101–103]. Properdin is thus likely needed to tip the balance to AP activation on cells that are well equipped with a repertoire of complement regulatory proteins. In the absence of intact complement regulators, as is also the case in aHUS, properdin might gain in function and promote the AP on healthy self-surfaces. Since absence of properdin is beneficial in these types of modeled diseases, the interesting question arises whether properdin blockage might also prevent the surface-directed complement activation in aHUS. Despite this attractive therapeutic potential, very few studies have been performed so far using experimental (animal or cellular) models of aHUS. Very recently, an important and interesting article was published showing that properdin inhibition in a mouse model of aHUS indeed improved disease outcome as expected [105].

In addition, properdin might have a role in exacerbating the thrombotic phenotype seen in aHUS. This is another reason why properdin blockage might be beneficial in this disease. Recent studies have elucidated a central role for properdin in the complement-mediated cross-talk between platelets and neutrophils. These two cell types mutually enhance each other’s activation, directly or indirectly via AP activation, and in doing so they are important mediators of thromboinflammation (extensively reviewed in [74]). First of all, activated platelets can act as a platform for local AP amplification. Properdin released from activated neutrophils can directly bind to the surface of activated platelets and can subsequently recruit the components needed for convertase formation to further promote complement activation on this platelet surface [67]. During this AP activation on platelets at sites of vascular injury, C5a is released which is a very important chemoattractant that can recruit and activate neutrophils. As previously explained, neutrophils can enhance their own activation in a positive feedback loop by secreting properdin that in an autocrine or paracrine fashion increases properdin-mediated AP activation on its own surface [80]. In turn, the released properdin can also promote AP-mediated platelet activation and complete the vicious cycle. In line with these findings, by using whole-blood ex vivo assays, Blatt et al. showed that blockage of properdin reduced platelet-leukocyte aggregate formation by 50% [47]. Furthermore, these studies collectively showed that the properdin-mediated mechanisms contributing to thromboinflammation were enhanced when the surface-protecting function of FH was experimentally inhibited [47, 67]. In disorders of compromised complement regulation such as aHUS, it has to be investigated if properdin blockage may therefore be beneficial in interrupting the positive feedback loops responsible for the increased (complement-mediated) thromboinflammation.

### Chronic proteinuric renal disease

#### Complement mediates proteinuria-induced tubulointerstitial injury

Glomerular dysfunction results in leakage of proteins through the glomerular filtration barrier. This proteinuria can induce tubulointerstitial injury and thereby is a strong predictor for the progression of chronic renal disease to end-stage renal disease [139, 140]. Complement activation at the surface of PTECs by filtered complement components has been proven to be a powerful mechanism underlying this proteinuria-induced tubulointerstitial injury (reviewed in [116, 117]). Under healthy conditions, complement components do not pass the glomerular filtration barrier, but in proteinuric patients complement proteins are found in the urine and deposition of complement along the tubular brush border (the apical side) is seen. Furthermore, complement inhibition attenuates the renal deterioration in proteinuric rodent models, confirming the role of complement in mediating tubulointerstitial injury [116, 117, 140].

#### The role of properdin in complement-mediated tubulointerstitial injury in proteinuria

It was hypothesized that properdin, as an initiator of AP activity, might play a role in triggering the tubular complement activation associated with tubulointerstitial injury in proteinuric renal disease [61]. Indeed, properdin was able to bind primary PTECs [61] and PTEC cell lines to act as a focal point for AP amplification followed by subsequent C3 and C5b-9 deposition [61, 141]. PTECs may be especially vulnerable to complement activation compared to other human cells because normally these epithelial cells do not come into contact with complement components [61] and express less complement regulatory proteins [142]. In addition, properdin was found in the urine of around 50% of proteinuric patients [143]. This urinary properdin associated with elevated sC5b-9 levels and with a worse renal function outcome but was not dependent on the degree of proteinuria [143]. Altogether, these findings advocate that properdin is an important determinant in intratubular complement activation and in this way it might play a role in the proteinuria-mediated renal damage.

#### The binding ligand for properdin on PTECs offers possibilities for specific therapeutic approaches

In subsequent studies, the binding ligand for properdin on renal tubular cells was identified: heparan sulfate proteoglycans. This type of proteoglycan is most abundant in renal tissues. It was found that properdin could bind specifically to certain heparan sulfate moieties of these proteoglycans in vitro and that properdin colocalized with the heparan sulfate proteoglycan syndecan-1 in vivo in proteinuric rat kidneys [68]. Interestingly, FH is also well-known for being able to bind to heparan sulfates; thus, it was speculated whether these positive and negative regulators might compete with each other in the tubular lumen under proteinuric conditions. Nonetheless, Zaferani et al. demonstrated that FH and properdin recognized different, non-overlapping epitopes of heparan sulfate chains on renal tubular epithelial cells, indicating at least no *direct* competition for binding sites [144].

In view of treatment strategies, this is an important and interesting finding. The authors have shown that certain low- or non-anticoagulant heparinoids could inhibit the interaction of heparan sulfates with properdin but not with FH, indicating that a specific blockage of the positive regulator properdin is feasible while leaving the inhibitory, beneficial actions of FH intact. Indeed, the in vitro experiments showed that these heparinoids inhibited C5b-9 deposition on the tubular epithelial cells and thus were able to control the AP of complement [144]. In summary, these findings illustrate that the composition of cell surfaces, and possibly their change during disease, may orchestrate the balance between AP activation and inhibition by recruiting properdin and/or FH.

## Future directions

### The potential of properdin as a therapeutic target in complement-mediated renal diseases

The interest in complement-targeted therapy has increased immensely in recent years, and many complement inhibitors are in the development pipeline [2]. Properdin is a relatively new kid on the block in the field of complement and immunity. Nonetheless, monoclonal antibodies against human properdin have been developed that are effective in blocking the AP in vitro [100, 145], and one anti-properdin antibody is currently at the stage of phase 2 in clinical trials [2]. Based on the role of properdin in promoting the activity of the AP, it is expected that properdin elimination can be effective in diseases of complement overactivation resulting in host tissue injury. To the best of our knowledge, no gain-of-function mutations in properdin itself have been found so far. Thus, therapy is aimed at compensating the complement dysregulation caused by other autoimmune or genetic factors (as in C3G and aHUS) or pathological conditions (as in chronic proteinuria).

In the previous sections, we have examined this potential of properdin elimination as a treatment for C3G, aHUS, and chronic proteinuric renal disease. Research so far has indicated that aHUS and chronic proteinuria might indeed benefit from properdin inhibition by limiting the properdin-mediated AP activation directed at host cell surfaces, i.e., the glomerular endothelium and proximal tubular epithelium, respectively. In addition, properdin inhibition in aHUS may decrease the thrombotic and inflammatory effects mediated by platelets and leukocytes. In contrast, properdin inhibition might *not* be a promising therapy in C3G, since properdin knockout in C3G mouse models clearly resulted in exacerbated renal injury. These findings might indicate that absence of properdin is advantageous in cases of uncontrolled tissue-bound activation, whereas in situations of uncontrolled fluid-phase activation, absence of properdin is unfavorable. This would be in line with the evidence for properdin being mainly a surface-directed regulator. C3G is not yet completely excluded as a potential disease that may benefit from properdin-targeted therapy. First of all, mice are not humans and the pathophysiological mechanisms may differ. The used mouse models were based on FH mutations, but such mutations are found in only a minority of C3G patients. C3NeF is a far more common abnormality found in C3G patients, and it has been shown that some C3NeF are dependent on properdin. Therefore, elimination of properdin to inhibit this C3NeF activity seems a plausible therapeutic option, but no studies on this subject have been published so far.

In summary, we are still at the beginning of understanding the effects of properdin-inhibiting therapy, and future research should help us to unravel properdin’s exact role in diverse disease settings. Its potential as a therapeutic target must be considered with care. One should always keep in mind the balance between the beneficial and detrimental functions that properdin can have in specific pathological settings. These differential effects are currently still hard to predict due to gaps in our knowledge about exact properdin biology, e.g., regarding the characteristics of the different oligomers, regarding the need of properdin for AP activation in different settings, and regarding the interaction of properdin with other immune effector molecules. Stratification of patient groups including a careful characterization of the specific AP defect and the clinical picture may aid in determining which patients may benefit from therapy. Other factors to keep in mind for safe and effective properdin inhibition are whether properdin should be inhibited locally or systemically and during which time window, i.e., acute or chronically.

### Properdin inhibition in comparison to C5 inhibition and its advantages

Eculizumab, a C5-inhibiting antibody, was approved in 2007 as the first complement-specific drug, and it is a very effective therapy for aHUS patients [146, 147]. It prevents C5a release and MAC formation and in this way reduces the subsequent complement-mediated damage to the endothelium. It was expected that eculizumab would also be effective in C3G, especially in patients showing increased C5b-9 levels. However, C3G patients show a heterogeneous response to eculizumab treatment [148, 149]. Given the previously discussed associations between properdin deficiency and decreased C5 convertase activity, properdin inhibition and C5 inhibition seem functionally similar to each other. Therapeutic inhibition of properdin therefore also seems promising in aHUS. Of note, both eculizumab use and properdin deficiency increase the susceptibility for meningitis. The prophylactic vaccination that is applied in patients receiving eculizumab [150] would therefore also be needed in properdin-blocking therapy.

In general, properdin-inhibiting therapy may have several advantages over C5 inhibition or over therapies directed to other complement proteins such as C3. First, in contrast to C5 inhibition, properdin inhibition may also reduce, at least to a certain extent, adverse inflammatory effects mediated by the upstream activation products C3a and C3b. Since properdin is present in much lower concentrations than C3 (and C5), it would also be a more manageable target to block [103]. Another important advantage of properdin inhibition is the specificity of blocking the AP, which is preferred in diseases showing a specific AP defect such as aHUS. C5 inhibition blocks all terminal pathway activity, whereas properdin inhibition only affects AP activity. The functioning of the other complement activation pathways is preserved so these can still fight infection and help maintain tissue homeostasis. This is supported by the study of Heinen et al., in which it was shown, using an in vitro enzyme-linked immunosorbent assay, that properdin blockage in normal human serum specifically impaired the ability to activate the AP but not the CP and LP [151]. Nonetheless, convincing experiments should be performed to confirm whether this also holds true for in vivo situations. It is not clear yet if properdin inhibition critically affects CP- and LP-induced complement responses by impairing the AP amplification loop, since the few studies performed on this topic contradict each other [47, 62, 74, 100, 145, 151]. These findings might also indicate that the role of properdin is context-dependent and may differ between disease settings. In line with this, it has been shown that the absolute requirement of properdin for AP activation depends on the activating surface; not all microbes need the presence of properdin to activate the AP [62]. If confirmed, not all AP-mediated actions will be compromised when properdin is inhibited. This indeed seems to be the case when looking at properdin-deficient individuals which specifically show increased vulnerability to *Neisseria* infections but not to others. In conclusion, compared to C3 inhibition or C5 blockage by eculizumab, properdin-directed therapy may provide a more sophisticated complement inhibition that does not completely compromise host defense but keeps some complement activation on several targets intact.

### Concluding remarks

Properdin is a recently discovered player in complement-related kidney diseases. We have just started to unravel its potential in therapeutics and further research is definitely needed. These studies should focus on increasing the understanding of properdin biology in general and its cross-talk with other immune pathways. Such research would benefit from validated and standardized quantitative assays for measuring properdin in serum or urine in well-defined patient groups. In combination with a diagnostic workup on other complement proteins, such data will provide us with important information on disease mechanisms in complement-mediated renal diseases and will help us to select patient groups that may benefit from properdin-directed therapy.

## Questions (answers are provided following the reference list)

1. Which statement about properdin is not true?
  - Properdin oligomerizes in dimers, trimers, and tetramers only upon stimulation
  - Properdin can recruit C3b and FB for new convertase assembly
  - Properdin stabilizes AP C3/C5 convertases, but not those of the classical and lectin pathway
  - Properdin oligomerization is essential for its function *in vivo*
2. Which factors should be taken into account when the function of properdin is studied:
  - Species differences
  - Aggregation of properdin upon freeze-thawing
  - The source of properdin: purified from serum, freshly released from cells, or in serum context
  - All of the above
3. Properdin is mainly produced by:
  - Hepatocytes
  - Leukocytes
  - Hepatocytes and leukocytes
  - Endothelial cells
4. Which of the following statements regarding the proposed role of properdin in renal disease is not true?
  - In proteinuria, properdin initiates and amplifies local complement activation on proximal tubular epithelial cells and thereby contributes to tubulointerstitial injury
  - Properdin prevents C5 conversion and thereby it has protecting roles in C3G
  - In aHUS properdin may both be involved in triggering the onset of disease as well as in exacerbating the thromboinflammatory course of disease
  - Properdin can be important for clearance of dangerous pathogens and altered self-cells, but its requirement for AP activation is not equal for all targets.
5. Which of the following statements regarding properdin-dependent and -independent C3NeF is true?
  - Properdin-dependent C3NeF are associated with a selective C3 consumption, which resembles the complement profile of FH^-/-^/P^-/-^ knockout mice
  - Properdin-independent C3NeF are associated with a selective C3 consumption and are most often found in C3GN
  - Properdin-dependent C3NeF are associated with elevated terminal pathway activation markers and are mainly found in C3GN
  - Properdin-independent C3NeF are associated with elevated terminal pathway activation markers and are most often found in C3GN

## Abbreviations

aHUS: Atypical hemolytic uremic syndrome
AP: Alternative pathway
C3G: C3 glomerulopathy
C3GN: C3 glomerulonephritis
C3NeF: C3 nephritic factor
CP: Classical pathway
DDD: Dense deposit disease
FB: Factor B
FD: Factor D
FH: Factor H
FI: Factor I
GBM: Glomerular basement membrane
LP: Lectin pathway
MAC: Membrane attack complex
PTEC: Proximal tubular epithelial cells
sC5b-9: Soluble C5b-9
TSR: Thrombospondin type 1 repeat
